# Myth or Modality? Exploring the current perceptions and practices of adrenaline use in musculoskeletal extremities surgery in Pakistan

**DOI:** 10.12669/pjms.41.8.12173

**Published:** 2025-08

**Authors:** Faaiz Ali Shah, Aimal Sattar, Haroon Ur Rashid

**Affiliations:** 1 Faaiz Ali Shah Orthopedics and Traumatology Division, Lady Reading Hospital, MTI, Peshawar Pakistan; 2 Aimal Sattar Orthopedics and Traumatology Division, Lady Reading Hospital, MTI, Peshawar Pakistan; 3 Haroon Ur Rashid Orthopedic Section Aga Khan University, Karachi Pakistan

**Keywords:** ACLA, Adrenaline, Digital Ischemia, Epinephrine, Finger Necrosis, Lidocaine, Local Anesthesia, WALANT, Xylocaine

## Abstract

**Objective:**

To get an overview of current surgical practices for determining the prevalence of myth that Adrenaline containing local anesthetic injections should not be used in acral surgeries because of potential for ischemic necrosis.

**Methodology:**

We conducted this descriptive cross-sectional study in Orthopedics and Traumatology Division Lady Reading Hospital MTI Peshawar Pakistan from January 10, 2025 to March 15, 2025. A validated 12 item questionnaire links in google form was sent via email to qualified Orthopedic surgeons who are members of Pakistan Orthopedic Association (POA). The questionnaire assessed the participants current clinical practice regarding frequency and patterns of Adrenaline Containing Local Anesthesia (ACLA) particularly in fingers and toes.

**Results:**

We sent 438 emails and received reply from 398(90.86%) participants. Majority (n=318, 79.89%) of the respondents admitted that they use injection Lidocaine (Xylocaine) with Adrenaline containing local anesthesia in their clinical practice. The top three areas for use were surgeries around the wrist (28.30%, n=90), shoulder (23.58%, n=75) and ankle (13.20%, n=42). Most (52.20%, n=166) of our participants still believed in myth and were of the opinion that ACLA should not be used in fingers and toes.

**Conclusion:**

The Orthopedic surgeons of Pakistan use injection Lidocaine (Xylocaine) with Adrenaline containing local anesthesia in their clinical practice more frequently in areas other than fingers and toes. Majority of the surgeons still persistently believe in Adrenaline myth and are of the opinion that this should not be used in finger and toe contrary to the reality supported by recent literature that it is completely safe in acral surgeries.

## INTRODUCTION

Adrenaline in combination with a local anesthetic (usually Lidocaine) is used in digital blocks to prolong the duration of local anesthesia and to create bloodless field for surgery thus avoiding use of tourniquet in extremity surgery. The preferred term used to describe this surgery now a days is denoted by the acronym WALANT which means Wide Awake Local Anesthesia No Tourniquet.^1^ The Adrenaline Myth was prevalent in 1925 to 1948 when surgeons believed that Adrenaline containing local anesthesia injection caused finger necrosis. For decades this Adrenaline dogma has rooted in traditional teachings in medical colleges where it was the usual teachings that do not inject Adrenaline in fingers, toes, nose, ears and penis.^2^ Later on evidence-based medicine revealed that the real culprit for necrosis and the source of myth was primarily the Procaine (Novocain) and not Adrenaline. The pH of Procaine was 3.6 and after prolonged storage and increased temperature it would further acidify resulting in very low pH of 1.

This acidity of Procaine rather than Adrenaline was the cause of finger necrosis.^3^ The use of Procaine was discontinued in 1948 when Lidocaine was introduced which is much safer. Moreover, the vasoconstriction effects of Adrenaline can be reversed with an alpha blocker antidote Pentolamine (although seldom used in clinical practice) introduced in 1949.^4^ Many large scale well designed studies reported that not even a single digit Adrenaline induced necrosis or loss has been documented uptil now and serves as bullets that had killed the myth of Adrenaline danger in fingers and toes.^5^,^6^ Although use of Adrenaline containing local anesthesia (ACLA) in fingers and toes has passed the initial phase of doubts in Western world after disclosure of safety results from numerous publications the myth has not yet clearly disproven universally.^7^ Surgeons from low middle-income countries like Pakistan still have persistent aversion to use Adrenaline containing local anesthesia in digital surgeries for fear of ischemia and possible litigation and this is still a strong taboo. The widespread of adoption of WALANT is therefore slow.^8^

The objective of our study was to get an overview of current surgical practices for determining the prevalence of myth that Adrenaline containing local anesthetic injections should not be used in acral surgeries because of potential for ischemic necrosis. The rationale of our study was that due to the paucity of local studies on this topic our study would address this critical gap in knowledge and would raise awareness of false historical official surgical dogma of Adrenaline containing local anesthesia in fingers and toes which seems persistently spans through generations and reinforced by older textbooks teaching and traditional teaching in medical colleges. Lidocaine with Adrenaline containing local anesthesia and WALANT has potential benefits to patients and because of the “Unfounded Surgical Dogma” the patients are deprived of this modality of treatment which could have been standard treatment for acral surgeries. The publication of this study would eliminate this misconception. This would bring about changes in surgical practice and motivate Orthopedic surgeons to use Adrenaline contacting local anesthesia without any fear as a standard of care and evidence-based treatment.

## METHODOLOGY

This descriptive cross sectional study was conducted in Orthopedics and Traumatology Division Lady Reading Hospital MTI Peshawar Pakistan from January 10, 2025 to March 15, 2025.The sample size for this study was calculated with the help of Solvin’s Formula^9^ proposed as n=N/1+Ne2 with “n” denoting sample size, “N” population size (1,253) and “e” margin of error(5%).Our sample size was 399.68 and by adding the possible non respondents rate of 10%(n=39) our final total sample size for this study was 438.

### Ethical approval:

The Institutional Review Board (IRB) Lady Reading Hospital Peshawar granted Ethical approval for conducting this study (Ref.No.29/LRH/MTI, dated January 9 2025).

All qualified Orthopedic surgeons registered with Pakistan Orthopedic Association (POA), working in Pakistan and consenting to participate in this study were included in our study. Orthopedic surgeons with incomplete data (valid email and cell number) were excluded. We got the data of all eligible Orthopedic surgeons from central office of POA Karachi. Stratified probability sampling technique was adopted for data collection by arranging all study participants province wise (Baluchistan, Khyber Pakhtunkhwa, Punjab and Sindh) and in alphabetical order with serial numbers. Through computer generated random numbers and using simple random technique with a minimum allocation proportion of 50% participants were selected from each province. An introductory email was sent to each participant briefly explaining the purpose of our study, asking for their consent to participate in this study and a link of questionnaire in google form. A soft reminder email was sent after two weeks to those who did not reply our initial email warning them that the questionnaire link would expire after four weeks.

We had followed all the mandatory steps for the development and validation of our study questionnaire. In the first step we sent our proposed study questionnaire and content validation Form along with instructions (for domains and items) to six senior Professors of Orthopedics for content validation and the Item-Level Content Validity(I-CVI) based upon their agreement was calculated (0.83). To clear confusion and further improve the items we distributed our questionnaire amongst 30 Orthopedic surgeons as pilot testing and based upon their suggestions we revised our questionnaire. We determined the consistency of our questionnaire by calculating the Cronbach’s alpha (0.8). Our final questionnaire had 12 questions with multiple choice answers as shown in Table-I.

**Table-I:** Our questionnaire of study.

S. No	Questionnaire items	Options		Tick √
1	Do you use injection Lidocaine (Xylocaine) with Adrenaline containing local anaesthesia in your clinical practice?	a	Yes	∗
		b	No	∗
2	If your answer is No, then what are the reason(s) for not using injection Lidocaine with Adrenaline containing local anaesthesia?	a	Risk of digital ischemic necrosis	∗
		b	Local Anaesthetic Systemic Toxicity (LAST)	∗
		c	Believe in surgical tradition of avoiding it in finger and toes	∗
		d	Other:	∗
3	For which purpose you use injection Lidocaine with Adrenaline containing local anaesthesia?	a	Vasoconstriction	∗
		b	Local Anesthetic	∗
		c	Both	∗
4	Where do you use injection Lidocaine with Adrenalin containing local anaesthesia?	a	Specify the area(s):	--------------
5	What do you think about the use of injection Lidocaine with Adrenaline containing local anaesthesia in fingers and toes?	a	It can be used safely in any case	∗
		b	It can be used in selective cases	∗
		c	It should not be used	∗
		d	Not sure about its use	∗
6	Why you prefer to use injection Lidocaine with Adrenaline containing local anaesthesia?	a	Current evidence	∗
		b	Personal experience	∗
		c	Traditional teaching in medical college	∗
		d	Not aware	∗
7	In last six months how many cases you did using injection Lidocaine with Adrenaline containing local anaesthesia?	a	No case	∗
		b	1 to 5 cases	∗
		c	6 to 10 cases	∗
		d	More than 10 cases	∗
8	In how many cases you used tourniquet?	a	No case	∗
		b	1 to 5 cases	∗
		c	6 to 10 cases	∗
		d	More than 10 cases	∗
9	How you completed your majority of cases using injection Lidocaine with Adrenaline containing local anaesthesia?	a	Completed the cases with one injection only	∗
		b	Completed the cases with two injections	∗
10	Overall how you evaluate the outcome in most of your cases using Lidocaine with Adrenaline containing local anaesthesia?	a	Very satisfied	∗
		b	Satisfied	∗
		c	Not satisfied	∗
11	What adverse effects or complications you have noticed while using injection Lidocaine with Adrenaline containing local anaesthesia?	a	Not at all	∗
		b	Other(Specify):	------------
12	What is your post fellow ship clinical experience?	a	Specify the duration:	-------------

### Statistical analysis:

We analyzed our data with SPSS version 28. All the quantitative variables of our study were expressed as Mean±Standard Deviation (SD) while qualitative variables were represented as frequencies and percentages. Chi square test, Independent Sample t test and One Way ANOVA tests were applied where appropriate for identification of any statistical association. P value <0.05 was considered to be significant.

## RESULTS

**Fig.1:**
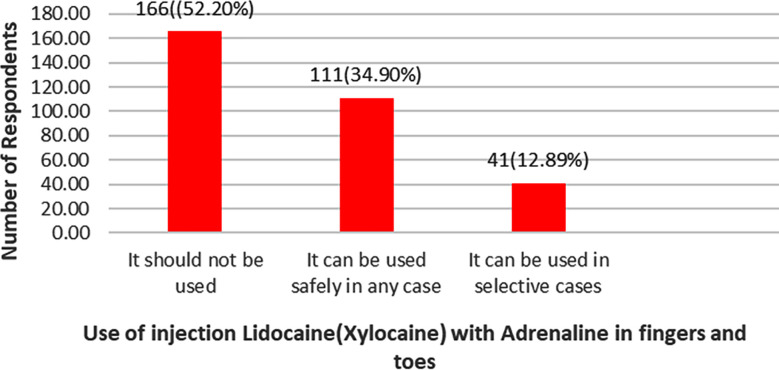
Bar graph showing views of respondents regarding use of Adrenaline containing local anesthesia in fingers and toes.

We sent the link of our study questionnaire in google Form through emails to 438 Orthopedic specialists. We received reply from 398(90.86%) participants while 40(9.13%) did not reply to our email. Majority (n=318,79.89%) of the respondents admitted that they use injection Lidocaine (Xylocaine) with Adrenaline containing local anesthesia in their clinical practice. A small percentage (20.10%, n=80) of the participants however admitted that they do not use it. Amongst the non-users of Adrenaline containing local anesthesia fear of risk of ischemia was the leading cause reported by 53(66.25%) participants followed by believe in surgical tradition of avoiding it in finger and toes (n=20,25%) and Local Anesthetic Systemic Toxicity (LAST) by 7(8.75%) participants. Most (87.73%, n=279) participants revealed that they used Adrenaline containing local anesthesia for the purpose of achieving both local anesthesia as well as vasoconstriction. In order of frequency the injection was reported to be used for surgeries around the wrist (28.30%, n=90), shoulder (23.58%, n=75), ankle (13.20%, n=42), arm (9.43%, n=30), thigh (7.54%, n=24), hip (6.91%, n=22), spine(5.06%, n=18), fingers and toes(5.34%, n=17). Regarding use of Adrenaline containing local anesthesia in fingers and toes, majority (52.20%, n=166) had the opinion that this should not be used as shown in Fig.1. About 140(44.02%) respondents preferred to use this combination based upon personal experience, 75(23.58%) use it based upon current evidence, 64(20.12%) traditional teaching in medical college and 39(12.26%) were not aware of the reason for its use. More than 10 cases were done using Adrenaline containing local anesthesia in the last six months by 170(42.71%) participants, one to five cases by 75(23.58%),6 to 10 cases by 58(18.23%), and no case by 15(4.71%) respondents. When inquired about the use of tourniquet, 189(59.43%) did not use tourniquet, 55(17.29%) participants used it in more than 10 cases, 42(13.20%) in one to five cases and 32(10.06%) used it in six to 10 cases.

Majority (90.25%, n=287) of the participants completed their cases with only one injection while 31(9.74%) participants admitted using two injections. Most (52.20%, n=166) participants were satisfied with the outcome of surgery using Adrenaline containing local anesthesia while 152(47.79%) were very satisfied. No adverse effects or complications were noted by 280(88.05%) participants. Tachycardia and circumoral numbness however were reported by 22(6.91%) and 16(5.03%) participants respectively. About 118(37.10%) study participants had post fellow ship clinical experience of more than 15 years, 90(28.30%) had 6 to 10 years, 57(17.92%) had 11 to 15 years and 53(16.66%) participants had one to five years post fellow ship clinical experience. We noted a statistically significant association(p=0.003) between greater clinical experience (>15 years) and greater number of cases (>15 cases) using Adrenaline containing local anesthesia, preferred to use it due to personal experience(p=0.02) and not using this combination in fingers and toes surgeries(p=0.01).

## DISCUSSION

In our study 79.89% (n=318) participants admitted that they use injection Lidocaine (Xylocaine) with Adrenaline containing local anesthesia in their clinical practice while 20.10% (n=80) were not using it. Fear of risk of ischemia was the leading cause of not using it as reported by 66.25% (n=53) participants followed by believe in surgical tradition of avoiding it in finger and toes by 25% (n=20) and Local Anesthetic Systemic Toxicity (LAST) by 8.75% (n=7) participants. Muscat et al. and Berner et al.^10^ reported that 42% respondents of emergency department and 100% of plastic surgeons use Adrenaline containing local anesthesia for scalp surgeries. About 5% emergency respondents and 23% plastic surgeons preferred Adrenaline containing local anesthesia for digital surgeries. Similarly, in another online survey^11^ amongst the South Australian doctors and medical students revealed that 80 respondents chose Adrenaline containing local anesthesia for excision of skin lesion on the back while only 10 respondents chose this for ring block(P<0.0001). These authors confirmed that the reason for few respondents opting for Adrenaline containing local anesthesia for ring block was because of the persistent myth of finger necrosis with Adrenaline.

Majority (52.20%, n=166) of our study participants were of the opinion that injection Lidocaine with Adrenaline containing local anesthesia should not be used in fingers in toes. About 140(44.02%) of our respondents preferred to use this based upon personal experience, 75 (23.58%) use it based upon current evidence, 64 (20.12%) traditional teaching in medical college and 39(12.26%) were not aware of the reason for its use. Similar to our study Deutsch et al. and colleague^8^ reported that 62.5% surgeons and 7.7% of nurses were least likely to use Adrenaline containing local anesthesia in fingers and toes. The participants of this study were reluctant to use Adrenaline containing local anesthesia in digital blocks because of fear of risk of digital ischemia. Majority (54%) of the participants admitted that this was thought to them in traditional teaching in medical college, 27% read this in books, 23% followed their senior colleague advice and 15% believed that this was their departmental policy not to use Adrenaline containing local anesthesia in digital blocks.

Our study participants used Adrenaline containing local anesthesia for surgeries around the wrist (28.30%, n=90), shoulder (23.58%, n=75) and ankle (13.20%, n=42). Majority (59.43%, n=189) of our respondents did not use tourniquet in their surgeries. Similar to our findings one study amongst 869 members of American Society for Surgery of the Hand (ASSH)^12^ documented that 62% members were using Adrenaline containing local anesthesia. Maximum (>60%) procedures performed with Adrenaline containing local anesthesia were surgeries on fingers without tourniquet. Only 4(2%) participants showed concerns of using Adrenaline in digital block.

In our study majority (88.05%, n=280) participants did not report any complications with Adrenaline containing local anesthesia. Tachycardia and circumoral numbness however were reported by 22(6.91%) and 16(5.03%) participants respectively. Greene et al. and Lalonde et al. reported “Adrenaline Rush” in 2.2%(n=8) and “Vasovagal Shock” in 1.8%(n=7) patients with Adrenaline containing local anesthesia. A systematic review^14^ reported complication rate of 1.7% with superficial skin infection being the most common. One randomized control trial conducted in Mayo Hospital Lahore Pakistan^15^ on 90 patients reported no complications of Adrenaline containing local anesthesia. Ilyas et al.^16^ reviewed the record of 4287 surgeries with Adrenaline containing local anesthesia and noted no complications. No recent evidence of casual relationship between the use of Adrenaline containing local anesthesia and necrosis of fingers or toes has been reported.^17^ Moreover no medico legal complaints against Adrenaline containing local anesthesia in France in 2007 to 2020 has been reported.^18^ To ensure optimal patient care we suggest targeted educational interventions like inclusion of this topic in Orthopedic residency curriculum to eliminate this discrepancy between evidence-based practice and clinical reality which exist in Pakistan.

Our study will greatly contribute to the medical literature as we had quantified the current prevalence of a persistent specific barrier (The Adrenaline Myth) to evidence based practice of Adrenaline containing local anaesthesia in finger and toes. Contrary to the robust evidence regarding safety of Adrenaline in fingers and toes in Pakistan, our study had identified a Knowledge-Practice Gap amongst the Orthopaedic surgeons of Pakistan. We believe that adhering to Adrenaline Myth will have negative consequences in terms of patient care and surgical practice as patients are devoid of an effective, safe and simple anaesthesia option. Strategies for bridging this gap should include updating undergraduate medical curriculum and Orthopaedic residency programs.

### Strength and limitation:

Our study had large sample size, a validated questionnaire and a higher response rate. To the best of our knowledge this will be the first study in Pakistan which will vanish the power of traditional teaching of “Never in fingers, nose, ears, and toes” Our study was restricted to one geographical area, Pakistan and this can be considered a limitation and hence limit the regional or global generalizability of our findings. We did not report patient’s preference for Adrenaline containing local anesthesia. Furthermore, we are unable to document departmental or institutional barriers to Adrenaline containing local anesthesia. We therefore recommend further studies to address all these limitations. We also suggest a Delphi process and organizing a National Consensus Conference supported by the associations of Orthopaedic surgeons and Plastic surgeons of Pakistan to develop evidence based national guideline for Adrenaline containing local anesthesia in acral surgeries.

## CONCLUSION

The Orthopedic surgeons of Pakistan use injection Lidocaine (Xylocaine) with Adrenaline containing local anesthesia in their clinical practice more frequently in areas other than fingers and toes. Majority of the surgeons persistently believe in Adrenaline myth and are of the opinion that this should not be used in finger and toe contrary to the reality supported by recent literature that it is completely safe in acral surgeries.

### Acknowledgement

This study does not involve any Personal, Financial or other Conflicts of Interest.

### Author’s contribution:

**FAS:** Did conception and design, questionnaire formulation, statistical analysis, editing and responsible for research integrity.

**AS:** Did Data collection and Manuscript Writing

**HUR:** Did study Questionnaire formulation and Validation, Review and final approval of manuscript.
